# Prevalence of Poultry Coccidiosis and Associated Risk Factors in Intensive Farming System of Gondar Town, Ethiopia

**DOI:** 10.1155/2019/5748690

**Published:** 2019-12-30

**Authors:** Anteneh Wondimu, Ermias Mesfin, Yehualashet Bayu

**Affiliations:** College of Veterinary Medicine, Haramaya University, P.O. Box 138, Dire Dawa, Ethiopia

## Abstract

A cross-sectional study was conducted from November 2018 to April 2019 in Gondar town, Ethiopia, with the objectives to determine the prevalence of coccidiosis and to assess the associated risk factors. The floatation technique was used for isolation of coccidian oocysts obtained from 384 fecal samples of chicken, and the prevalence revealed was 42.2%. The result showed 43.6% of male and 41.2% female chickens found infected with Eimeria. From the examined chickens, higher degree of infection was observed in the younger age group (51.0%) than adult chickens (36.7%). The difference was statistically significant (*p* < 0.05). The study showed relatively higher prevalence in poor body condition chickens (72.6%) than medium (36.1%) and good body condition (30.5%) with statistically significant difference (*p* < 0.05). The result also showed higher prevalence of coccidiosis in the floor system (50.4%) than in the cage system (19.0%), and the difference was statistically significant (*p* < 0.05). The prevalence based on the management system was 63.7%, 39.4%, and 29.3% in poor, medium, and good management, respectively. Significant difference was seen in the prevalence of poultry coccidiosis, between poorly and properly managed chickens (*p* < 0.05). In addition, the study reported 46.1%, 36.7%, and 26.3% prevalence in Bovan Brown, White Leg Horn, and Rhode Red Island chicken breeds, respectively. Coccidiosis is a major problem in the farm with inadequate hygienic measures and factors such as age, breed, body conditions, and biosecurity which are the most common factors that contribute for the occurrence of coccidiosis. Therefore, appropriate control strategies should be designed considering important risk factors such as, breed, age, management system, and housing system. Especially, focus should be given to biosecurity practices in the prevention and control of coccidiosis, and in addition, further studies are needed to be conducted to identify the prevalent Eimeria species for strategic control.

## 1. Introduction

In Ethiopia, in 2016/17, livestock population estimated 59.5 million cattle, 56.53 million poultry, 30.70 million sheep, 30.20 million goats, 8.44 million donkeys, 2.16 million horses, 1.21 million camels, and 0.41 million mules [1]. Though 1% of local birds was raised commercially in Ethiopia, reports in the year 2000 showed local birds contribute 98.5% and 99.2% of the total national egg and poultry meat production, respectively [2]. The traditional production system is characterized by minimum inputs; chickens gather foods, receive simple housing like sharing house with their owners at night, and breed naturally [3–5].

Coccidiosis is the most commonly encountered and economically important disease adversely affecting poultry production worldwide [6], and its prevalence and economic significance have been reviewed by different scholars in different production systems in Ethiopia [7–11]. Currently, poultry production are becoming more intensified which give greater chance in direct contact with their feces in a moist environment that ensures survival of the oocysts and their transmission from flock to flock.

Poultry coccidiosis is caused by single-celled protozoan parasites of the genus *Eimeria* and nine species [12, 13]. The most common and pathogenic species that affect the poultry is *Emeria tenella*, resulting in 100% morbidity and a high mortality [14]. In Ethiopia, *E. acervulina, E. necatrix, E. maxima,* and *E. tenella* are endemic in all parts of the country and affect mainly young growing birds [15].

The life cycle of all *Eimeria* species involves asexual (shizogony) and sexual phase (gametogony) which form oocyst [16]. Infection occurs by ingestion of sporulated oocyst, followed by the release of sporozoites in the duodenal lumen by mechanical and chemical action. The sporozoites invade the mucosa to start intracellular growth and asexual multiplication with periodic release of merozoites (develop to macro/male and micro/female gametes). Sexual phase occurs, fertilization of macrogamete by microgamete results in the formation of a zygote which is called as an oocyst [16]. The life cycle is rapid and may result in hundreds of thousands or even millions of oocysts produced from a single oocyst within 4 to 5 days [17].

The occurrence of clinical coccidiosis is related to several factors like host, agents, and environment interaction [13]. Detection of an oocyst in feces and postmortem examination of intestinal lesions are the most important procedures for the diagnosis of coccidiosis in chicken [18]. The economic importance of the disease associated with lost revenues; costs of treatments, vaccination/prevention, eradication, decontamination, and restocking [19, 20]. Despite the economic significance of the diseases in Ethiopia, in particular in the study area, no substantial research was done. Therefore, the study was conducted to investigate the current prevalence and associated risk factors of poultry coccidiosis in Gondar town poultry farms.

## 2. Materials and Methods

### 2.1. Description of Study Area

The study was conducted in Amhara regional state in Gondar town, Ethiopia. Gondar town is located 740 km Northwest part of Addis Ababa. The elevation of the area is about at latitude of 12 04'North, longitude of 27 02'East, and the altitude range between 1800-–2500 m.a.s.l. The climate of the region is somewhat warm with mean annual temperature of 20.50 C (17.2–23.90 C) and mean annual rainfall of about 1000 mm (600–1400 mm). Being a highland area, the town is spread on different mountains, slopes, and in valleys and has three small rivers, mainstreams, and a lake. The region receives a bimodal rainfall, the average annual precipitation rate being 1000 mm that comes from the long and short rainy seasons. The short rainy season occurs during March, April, and May, while the long one extends from June through September. According to the Office of Agriculture and Rural Development and Central Statistical Authority (2008) report, the livestock population in the area comprises of goat (22,590), donkey (9,001), cattle (8,202), sheep (2,695), horse (1,065), and unknown number of poultry [21]. Poultry production in the area characterized by cage, floor, and backyard production consists of local and exotic chicken breeds. Because of urbanization and land shortage, intensive production system became more popular in the study area.

### 2.2. Study Design and Population

A cross-sectional study was conducted from November 2018 to April 2019, on randomly selected exotic chickens breeds from six private farms located in Gondar town to estimate the prevalence and risk factors of poultry coccidiosis. A total of 384 faecal samples were collected during the study period. Farms selected purposively based on the higher chicken populations of the area as documented by the authorities of the Bureau of Agriculture. The information regarding age, breed, body condition, management, and housing type of chicken was gathered by short interview of owners. The study chickens were grouped into sex (male and female), breeds (White leghorn, Bovan Brown and Rhode Red Island), and ages classified as young (less than or equal to 2 months) and adult (older than 2 months) based on Oljira et al., [22]. Chickens were kept under floor and a cage husbandry system, and no medication given for the prevention of the disease in the farms including anticoccidial drugs except when chickens get sick.

### 2.3. Sample Size Determination and Sampling Method

To the best of literature review of the researcher, no previous documented works were conducted on prevalence of poultry coccidiosis in an intensive farm system in the study area. Therefore, in this study, sample size was determined depending on the expected 50% prevalence of coccidiosis, and 0.05 desired absolute precision according to Thrusfield formula [19].

(1) $$\begin{array}{c} N=\frac{1.96^{2}P\;\exp\left(1-P\;\exp\right)}{d^{2}}, \end{array}$$

where *N* = required sample size, *P* exp = expected prevalence, *D* = desired absolute precision, 1.96^2^ = *z*-value for 95% confidence interval. Therefore, 384 chickens were included in the research.

Based on the data from agriculture office, six private farms were selected considering higher number of chickens, and proportional samples were collected from each farms considering age, sex, breed, body condition, and housing type (Table 1). Sample collections were performed from individual animals using the simple random lottery method by applying dye for each chicken sampled to avoid double sampling. Around 10 samples of bottle per day were collected from each farm.

### 2.4. Faecal Sample Collection and Examination

Fresh faecal samples were collected by using a spatula from freshly voided faces and directly from the cloaca of selected chickens using spatula, and then the spatula was washed after each sample collection in order to avoid contamination. Each faecal sample was placed in a prelabeled bottle indicating the age (young and adult), breed (Bovans Brown, Leg Horn, and Rhode Red Island), sex (female and male), management (poor, medium, and good), body condition (poor, medium, and good), and housing method (floor and cage) and then transported to the University of Gondar Parasitology Laboratory for faecal examination. Samples were immediately stored in the refrigerator at 4°C until processed. The sample of an individual chicken was blended by a mortar and pistol, and then the floatation technique was applied using sodium chloride solution to harvest oocysts. Processed solution was poured through a tea strainer into a beaker then into a 12 ml or 15 ml centrifuge tube. The centrifuge tube was covered with a cover slip and allowed to stand for 10 minutes and then removed and placed on a slide and examined at 10x and then 40x magnifications to identify the oocyst [16].

### 2.5. Data Analysis

All collected raw data of the study were entered to a Microsoft Excel database system and imported to be analyzed using SPSS Version 20. Many attribute data imported to the database system includes type of production system, age, sex, breed, body condition, and laboratory result. Variation of infection prevalence between, age, sex, breed, body condition, and origin was determined by using chi-square (*χ*2) statistics. A *p* value of less than 0.05 at 95% confidence interval was considered as statistically significant.

## 3. Result and Discussion

The present study showed that poultry coccidiosis is an important health problem of chickens in the study area with overall prevalence of 42.2% (162/384). Compared with the results of previous researches, the present finding was lower than the reported prevalence (53.2%) in north Gondar in 2016 [7] and in Debre Zeit and Addis Ababa (48.2%) in 2001 [8]. This variation may due to variation in the geographical location, breed of chickens, management system, and anticoccidian drug usage. The present finding was higher as compared to 19.3% to 38.34% prevalence in different parts of Ethiopia [11, 14, 23–27]. In addition, the result of this research was very close to 43.6% prevalence reported in Gondar town [28]. The higher prevalence may be due to poor poultry management practices such as overcrowding, leaking water troughs, and faeces accumulation. The possible risk factors associated with the outbreak of coccidiosis in Ethiopia reported are absence of proper disposal of litters, wetting of litters from leaking pipes, and absence of all in all out system [10]. In addition, though no extensive study conducted in Ethiopia on anticoccidan drug resistance, the emergence of resistance has been documented to all compounds introduced to control coccidiosis in different parts of the world due to the extensive use of coccidiostats [10, 29, 30].

The study showed the prevalence of coccidiosis was relatively higher in younger age (51%) than adults (36.7%). The difference was statistically significant (*p* < 0.05) (Table 2). The result is consistent with 41%, with 18.3% prevalence in young and adult chickens, respectively [11]. But, it disagreed with Addis and Endale [26] and Temesigen et al., [31] who reported higher prevalence in adults. As reported in most published literatures, significantly higher prevalence of coccidiosis is observed in young than adult birds as earlier immunity is not well developed [17, 32, 33].

The prevalence of coccidiosis was relatively higher in male (43.6%) than female chicken (41.2%) (Table 2). This result is consistent with the previous studies who reported a higher prevalence of poultry coccidiosis in male than female chickens [28]. However, this result disagrees with high prevalence reports in females by different scholars [25, 26, 34], but the overall result showed not statistically significant difference (*p* > 0.05). This may be due to equal chance of both sexes for parasitic infection. Different researcher also reported that there is equal chance of coccidiosis infection between male and female chickens [35–37].

The effect of body condition on the disease prevalence showed that higher prevalence reported in poor body condition chickens (72.6%) than medium (36.1%) and good body condition (30.5%) (Table 2). This result agrees with works of different researchers in Ethiopia who reported higher prevalence in poor body condition [7, 26, 28]. This may be associated with highly exposed chickens, caused to have poor body condition as a result of low nutrient absorption and increased infection due to the decreased immunity that predispose for coccidiosis infection.

The prevalence based on breed showed 46.1%, 36.7%, and 26.3% in Bovan brown, White leg horn, and Rhode Red Island, respectively (Table 2). Relatively, this result is higher when compared with the results obtained from small- and large-scale farms in different parts of Ethiopia [38–40].

Higher prevalence was seen in the floor system (50.4%) than the cage system (19.0%) with statistically significant difference (*p* < 0.001) (Table 3). Similarly, Shubisa et al., [11] and Hadas et al., [28] reported higher prevalence in the floor system. This may be due to higher exposure of chickens to the sporulated Eimeria oocyst in the floor system. It is reported that environment such as overcrowding, leaking water troughs, and accumulation of faces are factors that contributed to the high prevalence of coccidiosis [10].

The prevalence based on the management system presented 63.7%, 39.4%, and 29.3% in poor, medium, and good management, respectively (Table 3). Significant difference was seen in the prevalence of poultry coccidiosis between poorly and properly managed chickens (*p* < 0.05). Similarly, reports showed coccidiosis is high in poor housing management [28]. Management of poultry houses plays a significant role in the spread of coccidiosis, because coccidian oocyst has high sporulation potential and easily spread in the poultry house environment [41].

Higher prevalence was also reported in Kes Gizachaw (65.7%) and W/ro Atseda (43.8%) farm (Table 4). This is may be due to a poor farm management system such as, farm location near to west disposable area, no biosecurity, and veterinary service. During the study period, disease outbreak was recorded in this farm causing the death of several chickens. Lower coccidiosis prevalence was reported in Tsilat (34.3%) and Salam and Sisay farm (32.6%), respectively. The lower prevalence may be due to a better management and biosecurity system.

## 4. Conclusion

The result of the current study showed poultry coccidiosis is the most common encountered and important disease affecting chicken production. The prevalence of coccidiosis was found low in poultry farms that practice high standard hygiene. High burden of coccidian infection was recorded in farms that were careless in observing adequate hygienic measures. Different putative risk factors have contributed for the occurrence of poultry coccidiosis infection in the study sites. Among these, age, breed, body conditions, and management system including a housing system and lack of effective biosecurity are the most common factors that contribute for the occurrence of coccidiosis. Therefore, biosecurity practices should be a primary focus in the prevention and control of coccidiosis. In addition, further studies needs to be conducted to identify the prevalent *Eimeria* species for strategic control.

## Figures and Tables

**Table 1:** Number of chickens in each farm and no. of the collected samples.

Farm name	No. of poultry	No. of examined
Tsilat	6000	172
BiruTesfa	2023	58
Selam and Sisay	3000	86
Asmellaw	510	17
Kes Gizachewu	1200	35
Atsede	480	16
Total	13,213	384

**Table 2:** Prevalence of coccidiosis based on chicken-related risk factor.

Risk factors	Categories	No. examined	No. of positives	Prevalence (%)	*χ*2 value	*p* value	OR	95% CI
Sex	Males	156	68	43.6	0.2	0.4	0.9	0.6–1.4
	Females	228	94	41.2				
	Total	384	162	42.2				

Age	Adult	237	87	36.7	7.6	0.004	0.6	0.4–0.8
	Young	147	75	51.0				
	Total	384	162	42.2				

Breed	RIR	38	10	26.3	1.2	0.2	1.6	0.6–3.8
	WLH	79	29	36.7	5.04	0.025	2.4	1.1–5.2
	BB^*∗*^							

Body condition	Medium	169	61	36.1	33.7	0.001	6	3.3–11.01
	Good	131	40	30.5	4.5	0.03	1.3	0.8–2.01
	Poor^*∗*^							

^*∗*^Used as reference, BB: Bovan Brown, WLH: White Leg Horn, RIR: Rhode Red Island.

**Table 3:** Environmental-related risk factor and prevalence of coccidiosis.

Risk factor	Categories	No. examined	No. of positives	Prevalence (%)	*χ*2 value	*p* value	OR	95% CI
Housing system	Cage	100	19	19.0	29.8	0.001	4.3	2.5–7.5
	Floor	284	143	50.4				
	Total	384	162	42.2				

Management	Medium	142	56	39.4	27.1	0.001	4.2	2.4–7.4
	Good	140	41	29.3	3.02	0.07	1.5	0.9–2.5
	Poor^*∗*^							

^*∗*^Used as reference.

**Table 4:** Prevalence of coccidiosis based on the farm site.

Farm name	No. of poultry	No. examined	No. of positives	Prevalence (%)
Tsilat	6000	172	59	34.3
BiruTesfa	2023	58	36	62.1
Selam and Sisay	3000	86	28	32.6
Asmellaw	510	17	9	52.9
Kes Gizachewu	1200	35	23	65.7
Atsede	480	16	7	43.8
Total	13,213	384	162	42.2

## Data Availability

Data used to support the findings of this study are available from the corresponding author upon request.
